# Patients' Experiences With Complete Tooth Loss and Conventional Complete Dentures Use: A Phenomenological Study

**DOI:** 10.1111/ger.70057

**Published:** 2026-02-14

**Authors:** Entisar Abdulkader, Richard Hovey

**Affiliations:** ^1^ Oral Health and Society, Faculty of Dentistry McGill University Montreal Quebec Canada; ^2^ Department of Restorative Dentistry, Faculty of Dentistry Sabha University Sabha Libya

**Keywords:** conventional dentures, edentulism, lived experience, qualitative research

## Abstract

**Objective:**

To explore the in‐depth experiences of individuals who have lived with edentulism and have long‐term experience with conventional complete dentures.

**Background:**

With the global rise in ageing populations, complete tooth loss (edentulism) and the subsequent use of dental prostheses have become increasingly common. Understanding the lived experiences of edentulous patients treated with conventional dentures is essential for dental professionals to provide personalised and empathetic care.

**Methods:**

This qualitative study employed an interpretive phenomenological analysis (IPA) approach. Completely edentulous participants with long‐term experience of using conventional complete dentures were recruited. Data were collected through semi‐structured, face‐to‐face interviews that were audio‐recorded and transcribed verbatim. Thematic analysis was used to identify key patterns and meanings across the participants' narratives.

**Results:**

Twelve participants expressed varied emotional and functional responses to edentulism. Some attributed their tooth loss to dental professionals, whereas others held themselves or their families responsible. Edentulism significantly affected oral function and social engagement. While several participants developed coping strategies to address the discomfort and limitations of conventional dentures, others perceived the dentures as beneficial, citing enhanced self‐image and improved chewing ability. Two main themes emerged: the long experience of tooth loss and experience of conventional complete dentures.

**Conclusion:**

This study contributes important insights for completely edentulous patients, conventional denture wearers and oral health practitioners into the complex emotional and functional dimensions of edentulism and denture use. The findings emphasise the value of patient‐centred care approaches that account for individual coping strategies, personal narratives and long‐term experiences with prosthetic rehabilitation.

## 1 Introduction

Edentulism, the total loss of natural teeth, remains a significant public health concern, profoundly affecting functional ability, nutrition, social participation and overall well‐being, particularly among older adults [1, 2, 3]. Although its prevalence has declined in many countries, the burden persists; in Canada, for example, approximately one in five older adults is edentulous [4]. Projections suggest that seniors will comprise 23.7% of the national population by 2036 [5], contributing to an increase in age‐related conditions such as tooth loss [4].

While often presumed to be an expected feature of ageing, edentulism reflects far more than biological processes alone. A growing body of literature demonstrates that patterns of complete tooth loss arise from a complex interplay of historical, social and structural influences [6, 7, 8]. These factors shape dental care experiences across the life span, positioning edentulism as a major life‐course transition, influenced by the interaction between individuals, their socioeconomic circumstances and dental care systems [1, 2, 8].

Cross‐national comparisons further illustrate this dynamic. In Canada, generational declines in edentulism have been attributed to improvements in preventive care, increased access to restorative dentistry and declining caries rates [4]. By contrast, countries with historically extraction‐oriented treatment cultures, such as New Zealand and Scotland, produced large cohorts of individuals who have lived most of their lives with complete dentures [8, 9]. In these contexts, present‐day oral health needs reflect professional norms and systemic structures shaped decades earlier.

Despite advances in preventive dentistry, a pathway towards irreversible full‐mouth extractions persists [10]. This trajectory reflects a clinical shift from preservation to replacement, often shaped by limited patient information and a constrained presentation of alternatives [11]. Effective clinician–patient communication is therefore essential, not only to establish a shared understanding of treatment risks and benefits, but also to support genuine adherence to tooth‐preserving options [12]. These findings highlight the need for upstream structural supports that enable truly informed, shared decision‐making. Within this context, dental clinicians play a pivotal role in helping patients navigate anxieties, manage expectations and develop coping strategies, reinforcing that prosthodontic care is inherently relational and psychosocial in nature.

Replacement of missing teeth with conventional complete dentures was considered the most common treatment choice in prosthetic dentistry. The consequences of this clinical pathway extend far beyond the functional limitations that conventional dentures aim to address [13]. Edentulism can profoundly disrupt self‐perception, social engagement, and quality of life, generating feelings of stigma, vulnerability and biographical disruption [11, 14, 15]. While conventional dentures restore appearance and some function, they also require ongoing physiological and psychosocial adaptation [16]. For instance, Fisk and colleagues reported that tooth loss can trigger a range of emotional responses from grief and reduced self‐confidence to social withdrawal and feelings of premature ageing [17]. Other qualitative studies have highlighted social impairments, elevated expectations about treatment planning, and a deep personal meaning attached to tooth loss and its management [11, 14, 16].

Although quantitative studies have documented functional outcomes and oral health–related quality of life among denture wearers, structured measures cannot fully capture the subjective and evolving nature of living with complete tooth loss [18, 19]. Qualitative research offers essential insights into these lived experiences, elucidating variations in adaptation, revealing the personal meaning of edentulism, and drawing attention to broader systemic influences, such as access to preventive care and institutional decision‐making processes that remain insufficiently addressed in dental research [20].

In this context, the present study sought to explore in depth the lived experiences of individuals with complete tooth loss, how they understand and interpret becoming edentulous, what it has meant to live with conventional dentures over time, and how these experiences shape daily oral function, identity, well‐being and social interactions.

## 2 Materials and Methods

### 2.1 Qualitative Approach

Interpretive Phenomenological Analysis (IPA) was adopted to explore the lived experiences of completely edentulous patients, as illustrated by Smith et al. [21]. IPA is a qualitative research approach developed for application in psychology, health, and the social sciences. It is grounded in phenomenological and hermeneutic traditions [21]. This method enables researchers to explore, examine, and interpret how participants make sense of their personal experiences [22, 23]. Moreover, IPA allows participants to express their narratives authentically, without imposed structure or modification [21]. Therefore, its adoption in this study places emphasis on uncovering the core essence of participants' lived experiences.

### 2.2 Research Participants

The aim of this study was to explore the phenomenon of complete tooth loss through the experiences of individuals who had lived with this condition. Consistent with the principles of phenomenological research, the focus was on the depth of experience rather than the number of participants [24, 25]. The study aimed to recruit 10–15 participants, aligning with recommendations for sample sizes in phenomenological research, which typically range from 2 to 25 participants [26]. A small, purposive sample was selected to enable the collection of rich, detailed data [21, 24, 25].

Included in the sample were English‐speaking male or female participants aged 18 years or older who were wearing a conventional complete denture and who were willing to participate and provide informed consent.

### 2.3 Recruitment Procedure

Participants were recruited from two cohorts of completely edentulous patients previously treated with conventional complete dentures at McGill University's Dentistry Clinic in Montreal, Quebec, Canada, between February 2017 and February 2019. Eligible participants were initially approached by an administrative staff member not involved in the study. Those who expressed interest had their contact information forwarded to a member of the research team, who provided additional information and addressed any questions.

A total of 12 completely edentulous individuals who met the inclusion criteria were enrolled. Within the context of an interpretive phenomenological study, a sample size of 12 is considered sufficient for in‐depth exploration of the phenomenon. Prior to providing written consent, the study was explained in detail to each participant to ensure mutual understanding of the research process and expectations.

### 2.4 Data Collection

Qualitative research utilises methods that provide access to participants' personal attitudes, concerns, behaviours, and lived experiences [27]. Given the nature of this study, face‐to‐face, in‐depth interviews were conducted between February 2018 and December 2019 by the same researcher (EA). All interviews were conducted in English at locations chosen by participants to ensure a relaxed and comfortable environment [28].

Participants were invited to discuss their experiences with complete dentures and the decision‐making process behind choosing this prosthetic treatment. A semi‐structured interview guide with open‐ended questions was used to facilitate detailed, narrative responses (Table 1). This format allowed participants to describe their thoughts and experiences in their own words while also enabling the researcher to probe topics that arose spontaneously [27]. Open‐ended questions also allowed the conversation to diverge naturally, offering deeper insight into themes that may not have been anticipated [29]. The interview guide, developed specifically for this study, began with general questions and gradually moved to more specific topics related to the research focus [30].

**TABLE 1:** Semi‐structured interview guide.

1. What does it mean to you to be completely edentulous?
2. Tell me about your experience of being edentulous. How about life since you lost your teeth?
3. What does it mean to live without teeth in your daily life and social life?
4. How did you manage to cope with all that?
5. How has living with this edentulous status affected your eating?
6. Let's talk about tooth replacement. When did it start and how was it?
7. How did you cope with the prosthetic rehabilitation process?
8. How would you describe your experience throughout the prosthetics treatment?
9. What foods do you usually like to eat? Are you able to eat the food you regularly eat?
10. What was the effect of prosthetic rehabilitation on your oral health?
11. How has living with complete dentures influenced your social life?
12. Has this affected other aspects of your life?
13. How did you cope with the treatment procedure or manage with all this?
14. Do you feel like your experiences with this denture have changed you as a person?
15. Is there anything else you would like to talk about or add?
16. What else would you like to share about your experience?

All interviews were audio‐recorded with participants' consent. Interview lengths ranged from 40 to 90 min. Audio recording is essential in qualitative research for accurate and complete data capture, allowing for verbatim transcription and reducing the risk of interviewer bias from note‐taking or memory recall. Transcripts included not only spoken words but also nuances such as pauses, laughter, and shifts in tone, providing richer data for analysis.

### 2.5 Data Analysis

The rationale for data analysis in Interpretive Phenomenological Analysis (IPA) is to gain an in‐depth understanding of participants' lived experiences of a specific phenomenon in order to address the research questions. Parahoo recommended that qualitative data be analysed directly after collection to preserve context and depth [31]. To ensure coherence, this study followed the IPA thematic framework proposed by Smith et al. [21]. This framework emphasises that researchers must immerse themselves fully in the data, becoming intimately familiar with each narrative to begin developing key interpretations and themes [32].

IPA allows researchers to explore how participants make sense of their experiences while also incorporating the researcher's interpretive role [32]. Initial interpretive analysis begins during data collection and helps guide subsequent interviews and sampling decisions. This iterative process ensures that the research remains responsive to emerging insights.

The analysis offered a deeper and richer understanding of the lived experiences of completely edentulous patients who were treated with conventional dentures. It also explored how meaning is constructed around this specific phenomenon within particular personal and clinical contexts [21].

In general, the IPA approach provides a flexible set of guidelines that can be tailored by researchers to fit their specific research objectives. The process involves several inductive and iterative stages, allowing for detailed and evolving engagement with the data. Emergent themes are developed through this process and serve to structure and support the interpretation and reporting of findings [33].

In practice, IPA provides a flexible but structured set of guidelines that can be adapted to the research aims. Analysis proceeded through several inductive and iterative stages, including initial noting, development of emergent themes, and the identification of connections across themes [33]. These stages supported the generation of a thematic framework that informed both interpretation and presentation of the findings.

Data collection and analysis occurred concurrently and continued until data saturation was achieved. Saturation was considered reached when no new insights were emerging from the interviews and when further coding no longer added meaningful analytic value [34]. [Correction added on 20 Feb 2026, after first online publication: The paragraphs under Section 2.5, “Data Analysis,” which had been duplicated in different wording, have been removed.]

### 2.6 Methodological Rigour and Reflexivity

To ensure the credibility and trustworthiness of the study findings, methodological rigour was a central component of this qualitative research [35, 36]. Reflexivity was integral to the research process, acknowledging that the researcher plays an active role in co‐constructing data and interpreting its meaning and requiring conscious, ongoing reflection throughout the study [37, 38].

All interviews were conducted by the main researcher, a dentist with clinical experience in patient care. This insider perspective provided a nuanced understanding of the clinical context and supported sensitive, empathetic engagement with participants; for example, familiarity with common challenges associated with complete denture use enabled the interviewer to recognise and explore participants' references to discomfort, instability, and adaptation without framing these experiences as treatment failure, thereby encouraging more detailed reflection.

Reflexivity was maintained through several strategies, including the use of a reflexive journal to document assumptions, preconceptions, and analytic decisions, and the completion of a reflexive form immediately after each interview to capture in‐the‐moment reflections (Appendix A). Regular discussions with the research team, including a supervisor with expertise in qualitative methodology, helped to identify potential biases, enhance interpretive transparency, and ensure analytic depth. Engagement with prosthodontic specialists further strengthened the accuracy and clinical relevance of the emerging findings.

Although the study involved a single interview with each participant, depth and authenticity were achieved through sustained engagement during each session, attentiveness to emotional cues, and a focus on exploring the meaning and significance of participants' experiences in detail. This approach ensured rich, nuanced data consistent with the interpretative aims of IPA. The study objective guided all stages of the research, from data collection to analysis. Together, the reflexive practices and methodological rigour employed in this study supported the credibility, transparency, and trustworthiness of the findings and strengthened the alignment between participants' lived experiences and the final interpretations.

## 3 Study Findings

### 3.1 Description of Participants

Twelve edentulous patients (four females and eight males) participated in the semi‐structured, open‐ended interviews. Participants ranged in age from 65 to 87 years and all had direct experience with complete tooth loss. The duration of edentulism among participants varied from 3 years to over 60 years.

All participants had been treated at McGill University's Faculty of Dentistry Dental Clinic in Montreal, Quebec, Canada, using a prosthetic treatment modality consisting of an upper conventional denture opposed by a mandibular overdenture. The duration of time living with this treatment ranged from 2 to 16 years.

All participants were retired at the time of the interviews; however, 3 were actively involved in volunteer work. 10 participants reported general health issues and were taking medications, while 2 were in good overall health.

Educational backgrounds varied, with most participants having completed at least secondary school, and some holding university degrees, as summarised in Table 2.

**TABLE 2:** Sociodemographic characteristics of participants.

Reference no.	Age (years), sex	Level of education	Years edentulous
P1	87 (F)	Limited education	27
P2	83 (M)	College	35
P3	75 (F)	College	45
P4	79 (M)	High school	25
P5	85 (F)	Secondary school	30
P6	86 (M)	High school	21
P7	78 (F)	College	27
P8	73 (M)	High school	69
P9	65 (M)	College	57
P10	73 (M)	College	25
P11	78 (M)	University degree	74
P12	75 (M)	University degree	71

To maintain confidentiality and protect participants' identities, they are referred to throughout the study by numerical codes (Participant 1–Participant 12).

### 3.2 Emergent Themes

Given the irretrievable nature of tooth loss, two superordinate themes emerged from the 12 participant interviews during the Interpretive Phenomenological Analysis (IPA) process. These themes are: (1) *the long experience of tooth loss* and (2) *the experience of wearing conventional complete dentures*.

Each superordinate theme includes several sub‐themes that capture the complex, diverse perspectives of the participants. These sub‐themes are explored within the context of their respective overarching themes, as illustrated in Table 3.

**TABLE 3:** Emergent themes/sub‐themes and key findings.

Main theme	Sub‐theme	Key findings
1. Long experience of edentulism	Meaning of tooth loss	Pain relief, regret, lack of awareness, poor hygiene
	Who's to BLAME	Self (neglect), Family (parental inattention), Clinicians (lack of advice)
	Impact of tooth loss	Loss of confidence, difficulty eating/speaking, social isolation
2. Experience with dentures	Positive experiences	Improved looks, confidence, speech, eating; long‐term satisfaction
	Negative experiences	Denture instability, pain, dietary limitations, social avoidance
	Coping strategies	Modifying food, avoiding eating out, using adhesives, psychological adaptation

#### 3.2.1 Long Experience of Edentulism

This superordinate theme captures participants' reflections on living without natural teeth, what edentulism has meant throughout much of their lives, whom they held responsible for their tooth loss, and the resulting impact on their oral functions and social experiences. Three key sub‐themes emerged from the data: (1) *meaning of tooth loss*, (2) *who to blame, and* (3) *impacts of tooth loss*.

##### 3.2.1.1 Meaning of Tooth Loss

When asked about the meaning of being edentulous, most participants described tooth loss as a result of a ‘lack of awareness’ and, for some, even ‘a relief from recurring dental problems’. Participants recounted poor oral hygiene practices earlier in life, which led to dental decay, periodontal disease, and ultimately the complete loss of their natural teeth.

Many participants admitted they did not brush regularly or practice proper oral hygiene, leading to progressive deterioration of their dentition. Their narratives reflected a lack of knowledge or emphasis on oral health maintenance:

I remember when I was young… I had so many problems with my teeth… I can tell there was no single tooth that was intact… one here needed to be filled and another one was moving and needed to be pulled out… with so much pain. I remember the horrible nights where I couldn't sleep. One day, I decided to go see a dentist to solve my problems… I ended up spending much of my time fixing them, but that didn't help. Honestly, I wasn't taking care of them at all… I didn't know I'd end up with false teeth, but I can say it's better than that pain and suffering. (Participant 7)

Several participants also reported that they did not realise the long‐term consequences of neglecting oral health until it was too late. They struggled with everyday tasks like eating or speaking due to deteriorating teeth, and social occasions were often stressful due to their dental condition:

I lost my teeth so many years ago… I haven't had teeth since I was about twenty‐seven years old… I was very young, I know that. I had dentures before I even got married… I didn't brush my teeth regularly, and they started decaying, one after the other. I realized that only later, when I had lost them all. (Participant 1)

In contrast, a few participants described tooth loss as a relief, an end to the chronic pain, dental procedures, and stress they had endured with their natural teeth. For them, dentures were not merely replacements but symbols of freedom from discomfort:

I can tell you frankly that it means nothing to be without teeth… It didn't bother me at all. I've lived most of my life with false teeth, and I had more problems when I had my natural teeth, one had to be removed, then another. I had toothaches all the time. When they finally removed everything, I felt more comfortable. It was a real relief not to have them. (Participant 5)

##### 3.2.1.2 Who's to Blame?

Losing natural teeth at an early age can have long‐term consequences for both oral and general health, as well as quality of life in later years. Preventing early tooth loss is a shared responsibility between patients and clinicians, reflecting the principles of person‐centred and preventive dentistry.

Among the study participants, views on responsibility for tooth loss varied. Some expressed a strong sense of *self‐blame*, acknowledging personal neglect and poor oral hygiene as contributing factors. Others assigned blame to *family members*, particularly parents, while several placed responsibility on *clinicians*, citing a lack of proper guidance and preventive intervention.

Participants who blamed *themselves* viewed their edentulism as the result of their own inaction, particularly failure to maintain oral hygiene or seek early intervention. They acknowledged that irregular brushing and a general disregard for oral care contributed to progressive tooth decay and eventual complete tooth loss:

I would say… losing my teeth is my own fault and I cannot blame anyone else… I was not following 100 percent hygiene control, as I did not brush my teeth regularly. Then I started to have decay in my front teeth, and I didn't care. I don't know, probably I was careless towards them. (Participant 8)

A few participants attributed their tooth loss to *family‐related* neglect during childhood. They reflected on growing up in large families where parents, especially mothers, had limited time and resources to manage each child's oral health. Lack of parental guidance around brushing, dietary habits, and dental visits were noted as contributing factors:

I could see that years ago, my mother had children one after the other… You know, she had so many children… she wasn't working, just busy with them… she had 12 children… oh, with no time to follow each one every single day… and by having too many children so close together… she didn't have time to take care of my teeth. (Participant 3)

The majority of participants expressed that *clinicians*, particularly dentists, had contributed to their tooth loss by complying with patient requests for extractions without offering sufficient education or alternatives. Many participants felt they were not adequately informed of the long‐term consequences of losing natural teeth, nor were they encouraged to pursue restorative options.

My top front teeth were not straight, and I looked ugly… I went to see my dentist and asked him to remove them because I didn't want them anymore. The dentist pulled the teeth without any hesitation. At that time, there was a lack of education and no motivation from the dentist's side. (Participant 4)

Another participant recalled a similar experience where dissatisfaction with her dental appearance led to irreversible decisions, made without professional counselling or resistance from the clinician:

I didn't like my teeth as they were crossed and big… and one… here… was black and chipped from tooth decay. I was working in a factory at the time and didn't have time to fix that ugly broken tooth. One day my sister said, ‘Go and fix them.’ Then I went to my dentist and asked him to take them all off, as I just wanted to get rid of them… He did what I asked him to do. He extracted them and offered me false teeth. (Participant 2)

##### 3.2.1.3 The Impact of Tooth Loss

Nearly all study participants reported experiencing struggles of varying degrees related to partial or complete tooth loss. These challenges extended beyond physical discomfort to affect their relationships, self‐confidence, and overall quality of life. The findings suggest that tooth loss had direct and profound implications on participants' self‐perception and personality.

For the majority of participants, living without teeth led to a marked decrease in self‐confidence and a negative change in how they perceived themselves. More than half expressed that tooth loss made it difficult for them to maintain a positive self‐image. As one participant explained:

I was much less self‐confident despite the successful things in my life… I was working with people and had a hard time living without teeth as it diminished my confidence. I was afraid of life situations because of my teeth. (Participant 6)

In addition to psychological effects, some participants experienced functional difficulties with eating and speaking, which further impacted their social interactions. Complete tooth loss impaired masticatory function for several individuals, causing disruptions in their social lives, especially when participating in gatherings with family and friends:

My social life was terribly disturbed; I was working and had to join other workers… I had to carefully choose foods I could manage to eat during lunch breaks. Normally, I was unable to eat out with my family and friends without teeth. (Participant 12)

Many participants also reported stress and embarrassment related to chewing difficulties in public, which led them to avoid certain foods or alter their diets to minimise discomfort. One participant recounted the impact on their professional life and social confidence:

When I started to lose my teeth because of gum disease, I was working as an engineer at a well‐known company here in Quebec. I felt stressed… It was not easy to live without teeth. I think when someone is working and is missing teeth or they look bad, eventually, you do not want to show them because everyone notices and might make comments. (Participant 2)

#### 3.2.2 Experience of Conventional Complete Dentures

The majority of the participants described the experience of teeth replacement with conventional denture as a life‐shifting experience. Hence, the main finding of ‘experience of conventional complete denture’ was identified with three sub‐findings that emerged from this main theme: (1) positive experiences, (2) negative experiences, and (3) coping strategies to overcome negative aspects of having conventional complete dentures.

##### 3.2.2.1 Positive Experiences

With the exception of two individuals, most participants expressed generally positive perceptions of their conventional dentures. Many interviewees described the experience as ultimately beneficial, contributing to improved self‐confidence, particularly in social settings.

One participant shared how dentures enhanced his self‐image and interpersonal interactions:

Once I had my dentures in my mouth, I felt better and I think I looked better, so I was more confident to sit down and talk to anyone else. (Participant 9)

The aesthetic improvement was particularly meaningful for some, who felt that dentures restored a sense of normalcy and attractiveness:

When I had a conventional denture and started living with it… I felt much better because when I smile there are teeth here, so cosmetically it feels better than an empty mouth. (Participant 11)

Beyond aesthetics, some participants noted functional improvements. Dentures allowed them to smile, speak, and eat more comfortably in social settings, as one participant explained:

I was not happy with the final results after years of using them, but I can say I was able to smile and speak with people… Not like when I was missing my teeth, because you know if you are missing a back tooth, it's not that big a problem like when you miss a front tooth… which is very evident. (Participant 8)

Although some initially found dentures uncomfortable, many adapted over time and were ultimately satisfied. Several credited a gradual transition from partial to full dentures as easing the adjustment:

I can say that the false teeth were just perfect… I had them for about seven years or more… They worked well, snapped right away, and were stable. I didn't expect that… and my denturist told me to be patient, but I had no issues. Maybe because I got them gradually, from a few missing teeth to a full set. (Participant 10)

Enhanced self‐esteem was also a common theme. Participants reported feeling more positive and socially accepted, regardless of how long they had worn dentures:

Actually, I feel more of a positive person now. It was probably the best thing that could ever have happened, to choose to get them… Everyone I speak with says that I seem to be happier and more relaxed with my new sets of dentures. (Participant 3)

##### 3.2.2.2 Negative Experiences

Despite positive feedback, several participants described negative experiences, particularly in relation to pain, denture instability, and dietary limitations. For some, unstable dentures caused trauma and persistent oral discomfort, leading them to remove the dentures frequently:

My dentures were never stable… oh… causing trauma to my gums and I had painful ulcers, and I used to take them out… It was always the lower one that caused this pain. (Participant 11)

As their oral tissues changed over time, some participants experienced increased difficulty with denture fit and function. This often led them to seek help from clinicians:

After months of pain and suffering from my dentures… they started moving after a few months… they were not staying in place anymore… then I went back to my denturist and asked him if he could change them or reline them, and he made new ones. (Participant 12)

Many also reported dietary modifications, as unstable dentures made it difficult to chew hard or sticky foods. These limitations disrupted social experiences and diminished the enjoyment of meals:

Carrot was my favorite food that I really enjoyed… but not anymore… I wasn't able to chew it with my false teeth… they wouldn't stay in place… I had to cut everything into small pieces, like steak or apples, or just say, ‘Sorry, I can't’, which wasn't always possible at events. (Participant 5)

For some, the inability to eat comfortably in public led to social withdrawal and a sense of embarrassment:

I was shy when talking to strangers, and I'm not a shy person at all… but I started to feel my teeth were loose and annoying. Dentures are not a good thing to start with… maybe they work for other people, but not for me. (Participant 7)

##### 3.2.2.3 Coping With Dentures

Despite these challenges, many participants developed coping strategies to manage their dentures over time. Dietary adjustments were the most common, with participants modifying or avoiding certain foods:

Food was a big problem and I had to make sure to mix my food in the mixer and cut my meat and the hard food into very tiny pieces to be able to eat it. (Participant 3)

Avoiding eating out was another coping strategy to reduce stress and embarrassment:

I avoided going out to eat, it was very rare. If I had to have a meal outside with family or friends, I wouldn't eat steak or anything hard. (Participant 12)

Some participants also employed cognitive strategies, reframing their experience and adapting to the situation with acceptance and self‐reliance:

I had my dentures for more than 30 years … I feel like they're part of my body and I had to get used to them… My mouth has changed and I don't have much gum left… I can manage with them when I forget they're there, and I also used to put stuff in (fixing materials) to manage, and it worked well. (Participant 4)

Overall, participants described conventional complete dentures as a major life change. While some were more satisfied than others, most employed a combination of practical and psychological strategies to adapt, including dietary changes, use of adhesives, and avoidance of social dining situations.

### 3.3 Overview of Phenomenological Findings

The findings of this study highlight a general lack of awareness among participants regarding the long‐term consequences of tooth loss and the critical role of oral hygiene in maintaining overall health and wellbeing. Many participants reported feeling a sense of relief after undergoing tooth extractions, especially during early adulthood, as this often resolved persistent dental pain or discomfort. However, this perceived benefit masked the long‐term risks and challenges associated with early and complete tooth loss.

The study also revealed that the use of conventional complete dentures brought mixed experiences. While many participants struggled with issues such as instability and discomfort, particularly with lower dentures, others reported significant improvements in self‐perception, social confidence, and the ability to eat and speak in public. Despite the difficulties associated with unstable dentures, participants demonstrated various coping strategies, including dietary adjustments, the use of adhesives, and psychological acceptance. These adaptations allowed them to regain a degree of normalcy and improve their quality of life.

Overall, the findings underscore both the negative and positive dimensions of living with edentulism and highlight the complex, evolving relationship that edentulous individuals develop with their prosthetic solutions over time.

## 4 Discussion

This interpretive phenomenological study explored the lived experiences of individuals who have lived without natural teeth for a significant portion of their lives. The use of open‐ended and semi‐structured interviews enabled participants to offer detailed and reflective accounts, revisiting earlier experiences, clarifying contextual details, and expanding on meanings across the interview, thereby generating the depth of data.

Two major themes emerged from narrative analysis: the long experience of tooth loss and the experience of conventional complete dentures. For each of the two findings, 12 completely edentulous participants explained their perspectives on their positive and negative experiences, which provide more insight into how tooth loss and prolonged denture use impact completely edentulous patients' quality of life.

The findings revealed that complete tooth loss held different meanings for participants, ranging from a lack of awareness to a sense of relief in living with non‐natural dentition. Most study participants were not aware of the possible consequences of tooth loss and wearing conventional complete dentures. Since many were unmotivated to maintain oral hygiene practices or had not been educated on how to preserve their natural teeth, they opted for tooth extraction at an early age to avoid further pain, discomfort, and other related complications. Information from this study's narrative data demonstrates that tooth loss can have dramatic implications on self‐perception and oral health functioning. Participants revealed how they experienced changes in self‐image and levels of self‐confidence. These findings are consistent with other qualitative studies related to tooth loss [10, 39]. Likewise, it has been suggested that the loss of any body part, including teeth, can lead to grief and changes in body image [40].

In this regard, Newton and Fiske explain that many patients relied on how clinicians communicate distressing health information, including news about their teeth [41]. Therefore, dental care providers needed to pay more attention to the emotional and psychological impacts of complete tooth loss on individuals and discuss the potential long‐term consequences on quality of life. Most of these implications might have been avoided with appropriate treatment plans; however, some of the research participants revealed that their dental clinicians chose to extract their natural teeth and provide them with removable dentures.

In contrast, some participants perceived tooth loss as a welcome relief from persistent dental problems and the ongoing burden of dental care. They described having more difficulties when they still had natural teeth, particularly due to tooth decay and periodontal disease that caused severe pain, repeated infections, and prolonged suffering. Managing these conditions required time and financial resources that many felt unable to sustain. As a result, some participants avoided discussing their dental issues with family members and instead sought clinicians who could remove their remaining teeth and provide conventional dentures. These narratives illustrate how tooth loss is intertwined with feelings of embarrassment, shame, and discomfort in social and family settings [11].

This pattern reflects broader concerns about care within increasingly privatised and fragmented dental systems, where preventive services are difficult to access and early interventions are inconsistently prioritised. The emotional labour required of clinicians to communicate the need for extensive tooth removal is substantial, and participants' experiences indicate that support during these critical decision‐making moments is often limited [41]. These observations underscore the need for patient‐centred communication and structured shared decision‐making frameworks within routine dental care.

The study also highlights the positive aspects of replacing missing natural teeth with conventional dentures. Replacement with conventional complete dentures remains one of the most common treatment options in prosthetic dentistry, helping restore oral function and aesthetics [42, 43]. Several participants described noticeable changes in their facial appearance caused by tooth loss and noted that dentures allowed them to eat more comfortably and smile in social situations. Trulsson and colleagues found that when edentulous patients were treated with fixed prostheses, they regained their social, psychological, and physical health and expressed strong gratitude for the prosthetic treatment [44]. In this study, participants perceived the benefits of conventional dentures positively, even though some were wearing lower overdentures at the time of data collection.

On the other hand, due to the nature of complete tooth loss, some participants were forced to change their diet to avoid difficulties caused by unstable conventional dentures. This dietary modification included avoiding hard and sticky foods. Similar findings were observed in a qualitative study conducted in the United Kingdom, which explored the impact of tooth loss and prosthetic rehabilitation on eating habits [45]. That study also found that participants were not always confident eating in public, although they did report improvements in functional ability and comfort when eating outside the home [45].

Despite the negative consequences of prolonged denture use, many study participants developed various coping strategies to manage problems related to unstable dentures. These strategies included modifying their diet and avoiding certain foods that were difficult to chew, especially in social settings. Edentulous patients generally felt more comfortable eating at home, where they could remove their dentures to clean away trapped debris, avoiding embarrassment during mealtimes [46].

Other participants developed cognitive coping strategies that helped them accept their dentures and mitigate emotional distress. Davis et al. noted that many denture‐related problems are often attributed to the condition of complete tooth loss itself, rather than the use of conventional dentures [11].

These findings have important implications for contemporary dental care. Participants' accounts reveal inconsistent application of preventive approaches earlier in their lives, with limited efforts to halt disease progression or support informed decision‐making before extraction. Strengthening preventive strategies across the life course, including affordability measures, improved access, enhanced oral health literacy, and sustained disease‐prevention programmes, is essential to reducing reliance on extraction‐driven pathways and ensuring that patients fully understand the consequences of tooth loss and the available alternatives.

The profound emotional impact of complete tooth loss further underscores the need for empathetic, patient‐centred communication, particularly when discussing extensive tooth removal. While existing literature often focuses on the emotional burden of partial edentulism [41], this study extends understanding by demonstrating the deeper and more enduring emotional consequences specific to total tooth loss. Using an interpretive phenomenological approach allowed these complexities to surface, highlighting how qualitative insights can inform more equitable and compassionate models of care.

Taken together, the findings show that complete tooth loss is not simply the result of individual choices but reflects cumulative life‐course inequalities, historical treatment norms, and the structural realities of dental care systems. Three implications emerge. First, the emotional and biographical disruption associated with total edentulism reinforces the urgency of comprehensive, life‐course preventive care. Second, gaps in the communication of treatment options, particularly when extractions are presented as the default or most affordable choice, point to the need for stronger shared decision‐making and clearer discussion of long‐term consequences. Finally, the study demonstrates the unique value of interpretive phenomenology for uncovering emotional, functional, and social dimensions of edentulism that remain obscured in quantitative research. These insights can guide more equitable oral health policies, strengthen preventive frameworks, and support compassionate, context‐sensitive prosthodontic practice for individuals living with the long‐term consequences of tooth loss.

## 5 Limitations and Strengths

To the best of the authors' knowledge, this is the first qualitative study to explore the lived experiences of edentulous individuals using conventional complete dentures in Montreal, Quebec, Canada. While the findings are grounded in a specific local context, they are likely transferable to similar populations across Canada and internationally.

A key strength of this study lies in the depth and richness of the data collected through face‐to‐face, open‐ended interviews. This approach allowed participants to share detailed narratives that offer meaningful insights into the long‐term experience of complete tooth loss and denture use. The inclusion of individuals who have lived with edentulism for decades adds further value to the findings, representing a unique and often underreported perspective.

Several limitations should be acknowledged. First, data collection involved a single in‐depth interview with each participant. Although this approach allows for rich idiographic accounts, it represents a departure from traditional Interpretative Phenomenological Analysis (IPA), which commonly incorporates multiple interviews over time to refine interpretations and engage participants in the iterative development and revision of themes. The absence of follow‐up interviews limited opportunities to revisit emerging interpretations, clarify meanings, and explore changes in participants' experiences over time. As a result, the findings should be understood as reflecting participants' perspectives at a specific point in time rather than as interpretations co‐constructed through prolonged engagement.

In addition, the relatively small sample size of 12 participants, while appropriate for an interpretive phenomenological approach, may limit the broader generalizability of the findings. Future studies involving larger and more diverse samples, as well as longitudinal or repeat‐interview designs, could further enhance understanding of how experiences with complete dentures evolve over time.

## 6 Conclusion

This study indicates that the experience of living with complete dentures is influenced by an interplay of functional outcomes, emotional responses, and social contexts. Although participants reported perceived improvements in function and appearance, ongoing challenges related to comfort, stability, and social confidence continued to affect their daily lives. Participants' accounts suggest that coping involved active processes of adjustment over time rather than simple acceptance of the prosthesis. From this perspective, edentulism can be understood as an experience that extends beyond technical rehabilitation and may require continued adaptation. These findings support the value of person‐centred approaches that recognise patients' experiences over time and emphasise education, expectation management, and follow‐up care to promote longer‐term adaptation.

## Author Contributions

Entisar Abdulkader, contributed to conceptualization, design, data collection, data acquisition, interpretation and analysis, drafted and critically revised the manuscript; Richard Hovey contributed to conceptualization, design, data acquisition, interpretation and analysis, critically revised the manuscript. All authors gave final approval and agreed to be accountable for all aspects of the work.

## Funding

The authors have nothing to report.

## Ethics Statement

This study has been ethically approved by McGill University's Institute of Research Ethics Broad Office. Signed consent forms were collected from all study participants and participants' anonymity and confidentiality were maintained.

## Conflicts of Interest

The authors declare no conflicts of interest.

## Data Availability

The data that support the findings of this study are available on request from the corresponding author. The data are not publicly available due to privacy or ethical restrictions.
